# Supplementary material for: End Sequence Analysis Toolkit (ESAT) expands the extractable information from single-cell RNA-seq data
Source: Genome Res. 2016 Oct;26(10):1397–410. doi: 10.1101/gr.207902.116 (PMC5052061; doi:10.1101/gr.207902.116)
Supplement: Supplemental Material [file supp_gr.207902.116_Supp_Materials.tar.gz › ESAT-esat_v0.1/doc/html/classbroad_1_1core_1_1primer3_1_1_primer_utils.html]

ESAT: broad.core.primer3.PrimerUtils Class Reference


ESAT


- Main Page
- Related Pages
- Packages
- Classes
- Files

- Class List
- Class Index
- Class Hierarchy
- Class Members

All Classes Namespaces Files Functions Variables Enumerator Pages

- broad
- core
- primer3
- PrimerUtils

Static Public Member Functions |
List of all members

broad.core.primer3.PrimerUtils Class Reference

|  |  |
| --- | --- |
| Static Public Member Functions | |
| static double | computeMaxTM (Alignment align) |
|  | |
| static double | computeTM (String seq) |
|  | |
| static void | main (String[] args) |
|  | |
| static double | percentGC (String seq) |
|  | |
| static double | computeTM (Alignment align) |
|  | |
| static double | computeTM2 (Alignment align) |
|  | |

## Member Function Documentation

|  |  |  |  |  |  |
| --- | --- | --- | --- | --- | --- |
| static double broad.core.primer3.PrimerUtils.computeMaxTM | ( | Alignment | *align* | ) |  |

static

Here is the call graph for this function:

|  |  |  |  |  |  |
| --- | --- | --- | --- | --- | --- |
| static double broad.core.primer3.PrimerUtils.computeTM | ( | String | *seq* | ) |  |

static

Here is the caller graph for this function:

|  |  |  |  |  |  |
| --- | --- | --- | --- | --- | --- |
| static double broad.core.primer3.PrimerUtils.computeTM | ( | Alignment | *align* | ) |  |

static

|  |  |  |  |  |  |
| --- | --- | --- | --- | --- | --- |
| static double broad.core.primer3.PrimerUtils.computeTM2 | ( | Alignment | *align* | ) |  |

static

Here is the call graph for this function:

Here is the caller graph for this function:

|  |  |  |  |  |  |
| --- | --- | --- | --- | --- | --- |
| static void broad.core.primer3.PrimerUtils.main | ( | String[] | *args* | ) |  |

static

Here is the call graph for this function:

|  |  |  |  |  |  |
| --- | --- | --- | --- | --- | --- |
| static double broad.core.primer3.PrimerUtils.percentGC | ( | String | *seq* | ) |  |

static

---

The documentation for this class was generated from the following file:

- java/broad/core/primer3/PrimerUtils.java


---

Generated on Fri Aug 15 2014 15:10:55 for ESAT by  

 1.8.7
